# A20 Attenuates Inflammatory Injury in Bovine Endometrial Epithelial Cells Through Autophagy-Mediated NLRP3 Inflammasome Inactivation

**DOI:** 10.3390/ani15243513

**Published:** 2025-12-05

**Authors:** Yongshuai Jiang, Fan Fei, Xiaoyu Wang, Yeqi Jiang, Kangjun Liu, Long Guo, Luying Cui, Heng Wang, Junsheng Dong, Jianji Li

**Affiliations:** 1 School of Basic Medical Sciences & School of Public Health, Faculty of Medicine, Yangzhou University, Yangzhou 225009, China; ysjiang0225@yzu.edu.cn; 2 Jiangsu Co-Innovation Center for Prevention and Control of Important Animal Infectious Diseases and Zoonoses, College of Veterinary Medicine, Yangzhou University, Yangzhou 225009, China; ffei00127@163.com (F.F.); souwang526@126.com (X.W.); jiangyq2025@163.com (Y.J.); yzdxlkj@163.com (K.L.); 18252712741@163.com (L.G.); lycui@yzu.edu.cn (L.C.); sdaulellow@163.com (H.W.); 3 Joint International Research Laboratory of Agriculture and Agri-Product Safety of the Ministry of Education, Yangzhou 225009, China; 4 International Research Laboratory of Prevention and Control of Important Animal infectious Diseases and Zoonotic Diseases of Jiangsu Higher Education Institutions, Yangzhou University, Yangzhou 225009, China

**Keywords:** A20, NLRP3 inflammasome, autophagy, LPS, BEECs

## Abstract

Endometritis, an inflammatory disease of the uterine endometrial tissue, is a major reproductive disorder in dairy cattle that causes extensive damage to endometrial epithelial cells. Excessive activation of the NLRP3 inflammasome is strongly associated with inflammatory pathology. Autophagy plays a critical role in clearing damaged proteins, organelles, and intracellular pathogens. Additionally, the zinc finger protein A20 exhibits potent anti-inflammatory effects across various inflammatory conditions. However, the roles of A20 and autophagy in regulating the NLRP3 inflammasome in BEECs remain poorly defined. This study shows that LPS significantly increased IL-1β expression, Caspase-1 activity, and lactate dehydrogenase (LDH) levels, while inducing numerous vesicular protrusions and membrane pores, resulting in severe inflammatory injury. A20 overexpression mitigated LPS-induced NLRP3 inflammasome activation and alleviated inflammatory injury. Conversely, autophagy inhibition or A20 silencing intensified LPS-induced NLRP3 inflammasome activation and inflammatory injury. Further analysis revealed that A20 promotes autophagy, and its inhibitory effect on the NLRP3 inflammasome was diminished when autophagy was suppressed. In conclusion, A20 reduces LPS-induced inflammatory injury in BEECs by enhancing autophagy and suppressing NLRP3 inflammasome activation. These results uncover a novel regulatory role for A20 in controlling excessive NLRP3 inflammasome activation in BEECs, suggesting its potential as a therapeutic target for bovine endometritis.

## Simple Summary

Bacteria commonly colonize the uterine cavity of dairy cows after parturition, frequently invading and infecting the endometrium, which leads to endometritis. This condition compromises endometrial function and contributes to elevated infertility rates. Bovine endometrial epithelial cells (BEECs), forming the first cellular barrier of the endometrium, represent the primary site of pathogen interaction and play a pivotal role in initiating innate immune responses. The NLRP3 inflammasome is essential for host defense against pathogens, yet its excessive activation can induce severe inflammatory injury. This study investigated whether the anti-inflammatory factor A20 modulates NLRP3 inflammasome activity in BEECs and explored the underlying mechanisms. We found that A20 overexpression attenuated LPS-induced NLRP3 inflammasome activation and reduced inflammatory injury. In contrast, A20 silencing exacerbated LPS-triggered inflammatory injury. Furthermore, A20-mediated suppression of the NLRP3 inflammasome was dependent on autophagy activation. Collectively, these findings indicate that A20 may serve as a promising therapeutic target for bovine endometritis and other NLRP3 inflammasome-related disorders.

## 1. Introduction

Postpartum uterine infections significantly affect dairy cow health, particularly in high-producing animals. Endometritis, one of the most common postpartum uterine diseases in dairy cows, is associated with reduced conception rates, extended calving intervals, increased insemination frequency, decreased milk production, and elevated culling rates [1,2]. Moreover, antibiotic treatment of bovine endometritis demonstrates limited efficacy in restoring fertility and overall health outcomes [3,4]. Consequently, developing novel therapeutic strategies for bovine uterine diseases is essential. Zinc finger protein A20, also known as Tumor Necrosis Factor α-Induced Protein 3 (TNFAIP3), functions as a key endogenous anti-inflammatory regulator [5]. It exerts protective effects in various inflammatory and autoimmune conditions, including inflammatory bowel disease, rheumatoid arthritis, keratitis, and systemic lupus erythematosus [6,7,8]. A20 acts as a negative feedback modulator of the NF-κB signaling pathway, exhibiting low basal expression under physiological conditions but rapidly upregulating during early inflammation. As a ubiquitin-editing enzyme, A20 effectively suppresses pro-inflammatory cytokine production and inhibits programmed cell death. Yu et al. demonstrated that A20 overexpression in corneal epithelial cells attenuates lipopolysaccharide (LPS)-induced inflammation, highlighting its therapeutic potential in ocular surface disorders [9]. In contrast, silencing A20 in gingival keratinocytes leads to significantly increased expression of inflammatory mediators and higher apoptosis rates [10]. Thus, A20 represents a promising target for mitigating inflammation and tissue damage in chronic inflammatory diseases.

The NLRP3 inflammasome plays a pivotal role in host defense against pathogenic microorganisms [11]. While moderate activation contributes to infection resistance and cellular homeostasis, excessive NLRP3 inflammasome activity can drive severe inflammatory pathologies such as inflammatory bowel disease, gout, and atherosclerosis [12,13,14]. This multiprotein complex, composed of NLRP3, ASC, and pro-caspase-1, is activated by Pathogen-Associated Molecular Patterns (PAMPs) or Damage-Associated Molecular Patterns (DAMPs). Upon assembly, it facilitates the autocatalytic cleavage of Pro-Caspase-1 into its active form (Cleaved-Caspase-1). The mature Caspase-1 then processes Pro-IL-1β into bioactive IL-1β (Cleaved-IL-1β), thereby initiating downstream inflammatory cascades [15,16].

Autophagy is a highly conserved intracellular degradation process that maintains cellular homeostasis by delivering damaged cytoplasmic components and organelles to lysosomes for turnover [17]. It critically regulates innate immune responses through recognition of PAMPs, elimination of intracellular pathogens, and modulation of inflammatory signaling pathways. Previous studies have shown that restoration of impaired autophagy suppresses LPS-induced NF-κB pathway activation in bovine endometrial epithelial cells (BEECs), leading to diminished inflammatory responses [18]. Accumulating evidence indicates that defective autophagy promotes aberrant NLRP3 inflammasome activation, resulting in excessive inflammation [13,19,20]. However, the mechanisms by which autophagy modulates NLRP3 inflammasome activity in BEECs remain incompletely elucidated.

Prior research has demonstrated that A20 can ameliorate fungal keratitis and inter-vertebral disc degeneration by enhancing autophagic flux [8,21]. In this study, we induced inflammatory responses in BEECs using LPS stimulation, manipulated A20 expression levels via lentiviral-mediated knockdown or overexpression, and suppressed autophagy either pharmacologically using specific inhibitors or genetically through ATG5 silencing via lentiviral transfection. These approaches were employed to investigate whether A20 regulates NLRP3 inflammasome activation through autophagy-dependent mechanisms. This study advances our understanding of A20’s role in modulating the molecular pathogenesis of bovine endometritis and identifies potential targets for preventive and therapeutic interventions.

## 2. Materials and Methods

### 2.1. Antibodies and Reagents

Antibodies against A20 (Cat# sc-166692, RRID: AB_2204516) and NLRP3 (Cat# sc-518122) were purchased from Santa Cruz Biotechnology (Dallas, TX, USA). Antibodies against p62 (Cat# 18420-1-AP, RRID: AB_10694431) and Caspase-1 (Cat# 22915-1-AP, RRID: AB_2876874) were obtained from Proteintech Biotechnology (Wuhan, China). Antibodies targeting ATG5 (Cat# 12994, RRID: AB_2630393), β-actin (Cat# 4970, RRID: AB_2223172) and the HRP-conjugated goat anti-rabbit antibody (Cat# 7074, RRID: AB_2099233) were purchased from Cell Signaling Technology (Danvers, MA, USA). The antibody targeting IL-1β (Cat# abs126104, RRID: AB_2890642) was obtained from Absin Bioscience (Shanghai, China). Antibodies targeting LC3 (Cat# M186-3, RRID: AB_10897859) and the HRP-conjugated goat anti-mouse antibody (Cat# 330, RRID: AB_2650507) were purchased from MBL Biotechnology (Beijing, China). LPS (L2880), DMEM-F12 medium (D8900), and pronase (P5147) were obtained from Sigma-Aldrich (St. Louis, MO, USA). Chloroquine (HY-17589A) was purchased from MedChemExpress (Shanghai, China). The reverse transcription kit 98 (AT341) and the SYBR Green qPCR Super Mix Kit (AQ601) were obtained from TransGen (Beijing, China). Fetal bovine serum (FBS, S711-001S) was purchased from Shuangru Biotechnology (Suzhou, China). The lactate dehydrogenase (LDH, A020-2-2) assay kit was obtained from Nanjing Jiancheng Bioengineering Institute (Nanjing, China). The FLICA 660 Caspase-1 assay kit (9122) was purchased from Immunochemistry Technologies (Davis, CA, USA). The Trizol reagent (DP424) was obtained from Tiangen (Beijing, China). Enhanced chemiluminescence kit (BMU102) was purchased from Abbkine (Wuhan, China). BCA assay kit (23227) was obtained from Beyotime (Shanghai, China). RIPA lysis buffer (C1053) and Protease inhibitor (P1265) were purchased from Applygen (Beijing, China). The lentiviruses for A20 overexpression and silencing, ATG5 silencing, and their respective negative control (NC) lentiviruses were obtained from Tsingke Biotechnology (Beijing, China). The short hairpin RNA (shRNA) target sequences were as follows: A20, GCACCGTGTTCGAAGGATACT; ATG5, GCAGTGGCTGAGTGAACATCT. The negative control shRNA sequence is TTCTCCGAACGTGTCACGT.

### 2.2. BEECs Isolation and Treatments

Primary BEECs were isolated and cultured according to established protocols [22]. Healthy uteri were collected from dairy cows at a local abattoir and immediately trans-ported to the laboratory on ice. The serosal layer of each uterus was disinfected with 70% ethanol, and a longitudinal incision was made to expose the endometrium. Uterine horns were then sectioned into 3–4 cm segments. Tissue blocks were washed with phosphate-buffered saline (PBS) containing 100 IU/mL penicillin and 100 IU/mL streptomycin before being placed in a 0.1% pronase digestion solution and incubated at 4 °C for 18 h. The endometrial epithelium was subsequently scraped off using a sterile blade and collected in PBS. After centrifugation at 100× *g* for 5 min, the resulting cell pellet was resus-pended in DMEM/F-12 medium supplemented with 10% fetal bovine serum and 100 IU/mL penicillin/streptomycin, and cells were maintained at 37 °C under 5% CO_2_. Cells were passaged upon reaching 90% confluence for use in subsequent experiments.

To examine the role of autophagy in inflammation, autophagy was inhibited either by pretreatment with 20 μmol/L chloroquine (CQ) for 4 h or via lentiviral transfection with shATG5 (with non-targeting shRNA serving as negative control, NC). Lentiviral transduction was also used to generate A20 overexpression (OE-A20) or knockdown (shA20) cell models to assess the impact of A20 on inflammatory responses and autophagy, with corresponding non-targeting controls used as NC. An inflammatory injury model was established by stimulating BEECs with LPS (1 μg/mL) for 24 h.

### 2.3. LDH Release Assay

LDH, an indicator of cell membrane permeability, was measured to assess inflammatory damage induced by lipopolysaccharide (LPS) in BEECs. LDH levels in the culture supernatant were quantified using a commercial detection kit. Following treatment, the supernatant was collected, centrifuged to remove debris, and transferred to a 96-well plate. Absorbance was then measured at 440 nm with a microplate reader (Multiskan FC, Thermo Fisher Scientific, Waltham, MA, USA).

### 2.4. Scanning Electron Microscopy (SEM)

Cells were cultured on glass coverslips placed in 24-well plates. Following treatment, the coverslips were rinsed with PBS and fixed with 4% glutaraldehyde for 2 h in the dark. Subsequently, samples were post-fixed with 1% osmium tetroxide at 4 °C for 2 h. After an-other PBS rinse, samples were dehydrated through a graded ethanol series and subjected to critical point drying. The dried specimens were then sputter-coated with gold. Morpho-logical changes in the cell membrane were examined using SEM (GeminiSEM 300, ZEISS, Oberkochen, Germany).

### 2.5. Caspase-1 Activity Assays

The treated cells were collected and incubated with the fluorescent inhibitor probe FLICA 660-YVAD-FMK (Immunochemistry Technologies, Davis, CA, USA), which specifically labels activated Caspase-1, for 1 h at 37 °C in the dark. Stained cells were subsequently analyzed using flow cytometry (FACSVerse, BD Bioscience, San Jose, CA, USA).

### 2.6. Quantitative Reverse Transcriptase-PCR (qRT-PCR)

Total RNA was extracted from treated BEECs using TRIzol reagent (Tiangen, Beijing, China), and cDNA was synthesized from 900 ng of total RNA using the reverse transcription kit. QRT-PCR was performed using the SYBR Green qPCR Super Mix Kit on a CFX Connect Real-Time PCR system (Bio-Rad, Hercules, CA, USA). The housekeeping gene β-actin served as an endogenous control. The PCR conditions were: 94 °C for 30 s, followed by 45 cycles of 94 °C for 5 s and 60 °C for 30 s. The relative expression levels of mRNA were quantified using the 2^−ΔΔCt^ method. The primer sequences for the target genes are listed in Table 1.

### 2.7. Western Blotting

Total protein was extracted from treated BEECs using RIPA lysis buffer supplemented with protease inhibitors. Protein concentrations were determined by the BCA protein assay. Equal amounts of total protein (30–60 µg) from each group were separated via 10–12% SDS-PAGE and transferred onto PVDF membranes. Membranes were blocked with 5% non-fat milk at room temperature for 1 h, followed by overnight incubation at 4 °C with primary antibodies targeting A20 (1:200), IL-1β (1:1000), NLRP3 (1:500), Caspase-1 (1:2000), LC3 (1:1000), p62 (1:5000), ATG5 (1:1000), and β-actin (1:1000). After TBST washes, membranes were incubated with corresponding HRP-conjugated goat anti-rabbit IgG (1:2000) or anti-mouse IgG (1:5000) for 1 h at room temperature. Following TBST washes, protein bands were visualized using an Enhanced Chemiluminescence detection system (5300, Clinx, Shanghai, China). Band intensities were quantified using ImageJ software (ImageJ 1.53q, National Institutes of Health, Bethesda, MD, USA).

### 2.8. Statistical Analyses

Data were obtained from three independent replicates. Statistical analyses were con-ducted using SPSS 17.0 and GraphPad Prism 6.0. Quantitative data are expressed as mean ± standard error of the mean (SEM). One-way ANOVA followed by Tukey’s multiple comparisons test was applied for group comparisons. A *p*-value < 0.05 was considered statistically significant.

## 3. Results

### 3.1. LPS Induced Inflammatory Injury in BEECs

**Figure 1:**
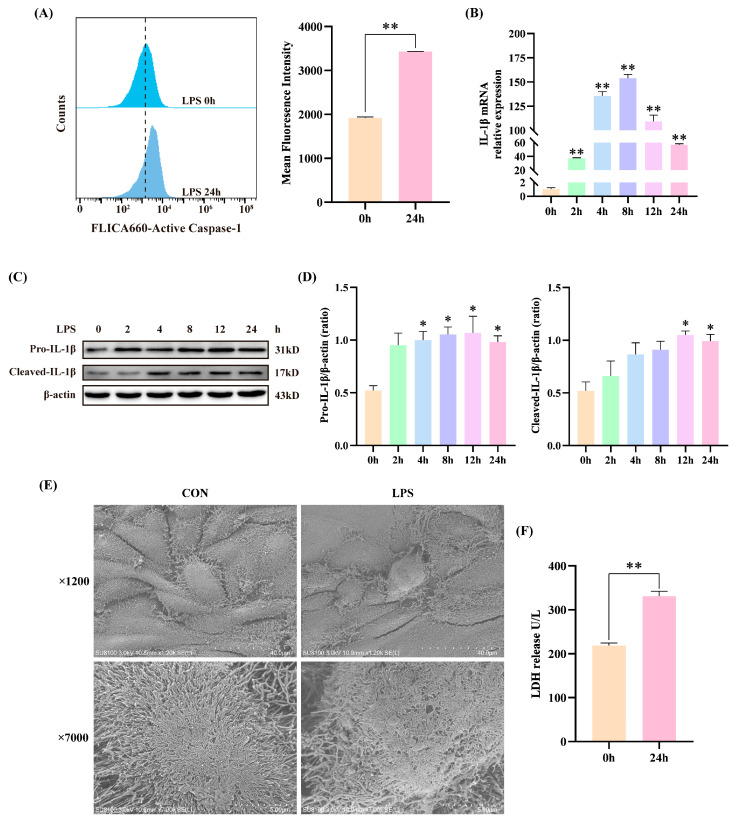
LPS induced NLRP3 inflammasome activation in BEECs. (**A**) Analysis of active Caspase-1 using flow cytometry. (**B**) The mRNA expression levels of IL-1β measured by qRT-PCR. (**C**) Protein expression levels of Pro-IL-1β and Cleaved-IL-1β measured by Western blot. (**D**) Quantitative analysis of Pro-IL-1β and Cleaved-IL-1β. (**E**) Membrane changes in BEECs observed using SEM. Magnification: 7000× and 1200×. (**F**) LDH release detected in the supernatant. Data are presented as the mean ± SEMs of three independent experiments. * *p* < 0.05, ** *p* < 0.01 vs. LPS treatment at 0 h.

Activation of Caspase-1 and maturation of IL-1β are characteristic features of NLRP3 inflammasome activation, and excessive activation can lead to inflammatory damage. In this study, BEECs were treated with LPS, and cell lysates were collected to assess Caspase-1 activity using flow cytometry, while IL-1β expression was evaluated by qRT-PCR and Western Blot. The results demonstrated that LPS activated Caspase-1, increased IL-1β mRNA expression, and elevated the protein expression of Pro-IL-1β and Cleaved-IL-1β (Figure 1A–D). Cell membrane rupture and LDH release induced by inflammatory damage were assessed using SEM and an LDH kit, respectively. Figure 1E revealed that LPS caused pronounced pores and numerous blister-like protrusions on the cell membrane compared to the control group (0 h treatment with LPS). Additionally, LDH levels in the cell supernatant were significantly elevated (Figure 1F). These results indicate that the NLRP3 inflammasome in BEECs is activated by LPS, resulting in cellular inflammatory damage.

### 3.2. Inhibition of Autophagy Enhanced LPS-Induced Activation of the NLRP3 Inflammasome

**Figure 2:**
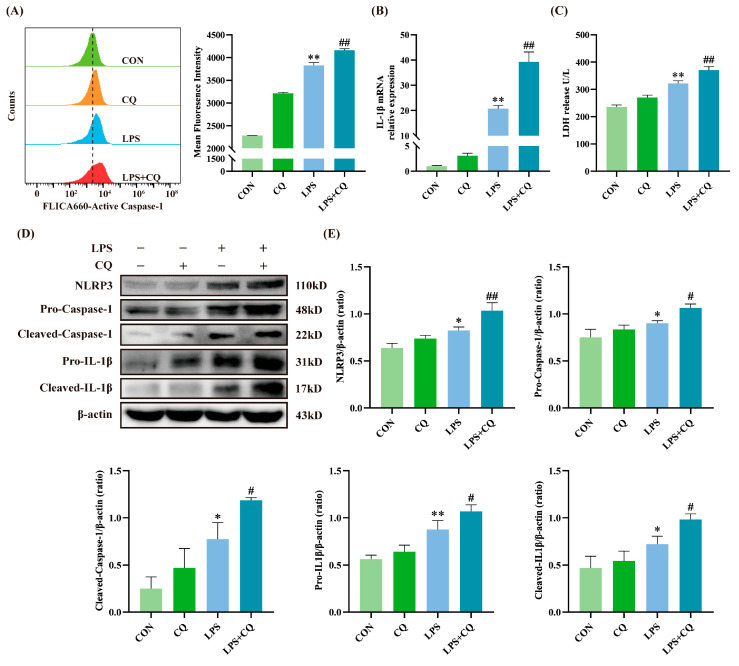
Inhibition of autophagy by CQ exacerbated LPS-induced inflammatory damage in BEECs. (**A**) Analysis of active Caspase-1 using flow cytometry. (**B**) The mRNA expression levels of IL-1β measured by qRT-PCR. (**C**) LDH release detected in the supernatant. (**D**) Protein expression levels of NLRP3, Pro-Caspase-1, Cleaved-Caspase-1, Pro-IL-1β, and Cleaved-IL-1β measured by Western blot. (**E**) Quantitative analysis of NLRP3, Pro-Caspase-1, Cleaved-Caspase-1, Pro-IL-1β, and Cleaved-IL-1β. Data are presented as the mean ± SEMs of three independent experiments. * *p* < 0.05, ** *p* < 0.01 vs. the control (CON) group, and # *p* < 0.05, ## *p* < 0.01 vs. the LPS group.

To examine whether autophagy regulates the NLRP3 inflammasome in BEECs, autophagy was inhibited using the autophagy inhibitor CQ, followed by LPS treatment. As demonstrated in Figure 2A–C, CQ enhanced Caspase-1 activity, IL-1β mRNA expression, and LDH release compared to the LPS group, suggesting that autophagy inhibition aggravated inflammatory injury in BEECs. LPS alone increased the expression levels of NLRP3, Pro-Caspase-1, Cleaved-Caspase-1, Pro-IL-1β, and Cleaved-IL-1β. Moreover, CQ-mediated autophagy inhibition further enhanced the expression of these proteins (Figure 2D,E).

**Figure 3:**
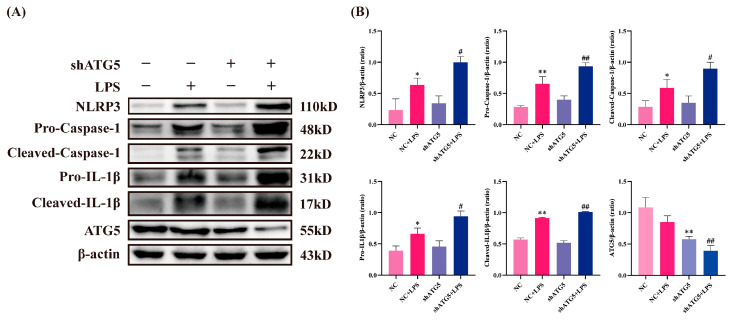
Silencing ATG5 inhibited autophagy and exacerbates LPS-induced activation of the NLRP3 inflammasome in BEECs. (**A**) Protein expression levels of NLRP3, Pro-Caspase-1, Cleaved-Caspase-1, Pro-IL-1β, Cleaved-IL-1β, and ATG5 measured by Western blot. (**B**) Quantitative analysis of NLRP3, Pro-Caspase-1, Cleaved-Caspase-1, Pro-IL-1β, Cleaved-IL-1β and ATG5. Data are presented as the mean ± SEMs of three independent experiments. * *p* < 0.05, ** *p* < 0.01 vs. the NC group, and # *p* < 0.05, ## *p* < 0.01 vs. the NC + LPS group.

ATG5 was silenced via lentiviral transfection to reduce autophagic activity and further confirm autophagy’s role in BEECs NLRP3 inflammasome activation. The transfection successfully decreased ATG5 expression levels. ATG5 silencing significantly enhanced the LPS-induced increase in expression levels of NLRP3, Pro-Caspase-1, Cleaved-Caspase-1, Pro-IL-1β, and Cleaved-IL-1β (Figure 3). These results indicate that autophagy inhibition exacerbates LPS-induced NLRP3 inflammasome activation, thereby intensifying inflammatory injury in BEECs.

### 3.3. A20 Promoted Autophagy in BEECs

**Figure 4:**
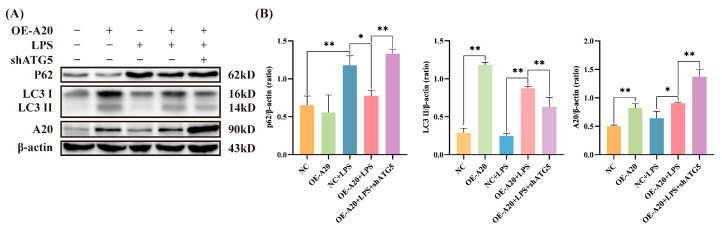
A20 enhances BEECs autophagy. (**A**) Western Blot detection of changes in key autophagy proteins P62 and LC3II expression. (**B**) Quantitative analysis of P62, LC3II, and A20. Data are presented as the mean ± SEMs of three independent experiments. * *p* < 0.05, ** *p* < 0.01.

To determine whether A20 overexpression promotes autophagy, Western blotting was performed to detect the autophagy markers LC3 and p62. As shown in Figure 4, A20 overexpression via lentiviral transduction increased the expression levels of A20 and LC3II, while p62 expression decreased compared to the control group, demonstrating that A20 promoted autophagy in BEECs. LPS treatment alone reduced LC3II expression and increased p62 expression; however, A20 overexpression reversed these changes, resulting in elevated LC3II and decreased p62 levels. These findings confirm that LPS inhibits BEECs autophagy, while A20 enhances autophagy and counteracts LPS-induced autophagy impairment.

### 3.4. A20 Alleviates LPS-Induced Inflammatory Injury

**Figure 5:**
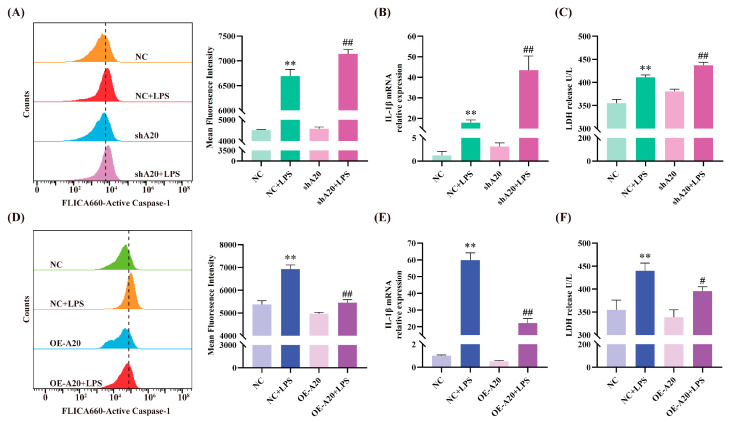
A20 attenuated LPS-induced inflammatory damage in BEECs. (**A**,**D**) The analysis of active Caspase-1 using flow cytometry. (**B**,**E**) The mRNA expression levels of IL-1β measured by qRT-PCR. (**C**,**F**) The release of LDH detected in the supernatant. The data were presented as the mean ± SEMs of three independent experiments. ** *p* < 0.01 vs. the NC group, and # *p* < 0.05, ## *p* < 0.01 vs. the NC + LPS group.

To investigate whether A20 regulates BEECs inflammatory injury, cells were transfected with lentiviruses carrying A20 overexpression or silencing plasmids. As shown in Figure 5, LPS stimulation increased Caspase-1 activation, IL-1β mRNA expression, and LDH release. A20 silencing further aggravated these changes, while A20 overexpression attenuated LPS-induced increases in Caspase-1 activity, IL-1β mRNA expression, and LDH release. These results demonstrate that A20 inhibits LPS-induced BEECs inflammatory injury.

### 3.5. A20 Reduced NLRP3 Inflammasome Activity via Autophagy

**Figure 6:**
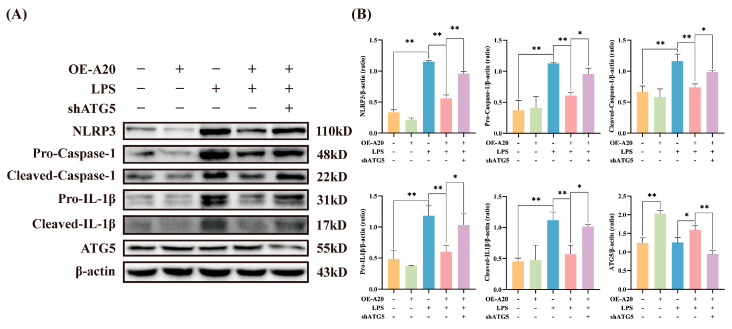
Inhibiting autophagy in BEECs weakened the effect of A20 on suppressing the activation of the NLRP3 inflammasome. (**A**) The protein expression levels of NLRP3, Pro-Caspase-1, Cleaved-Caspase-1, Pro-IL-1β, Cleaved-IL-1β, and ATG5 measured by Western blot. (**B**) Quantitative analysis of NLRP3, Pro-Caspase-1, Cleaved-Caspase-1, Pro-IL-1β, Cleaved-IL-1β and ATG5. The data were presented as the mean ± SEMs of three independent experiments. * *p* < 0.05, ** *p* < 0.01.

To further elucidate the potential mechanism of A20 in inhibiting inflammatory injury, BEECs were transduced with lentivirus to overexpress A20 and silence ATG5. As shown in Figure 6, A20 overexpression significantly inhibited the expression of NLRP3, Pro-Caspase-1, Cleaved-Caspase-1, Pro-IL-1β, and Cleaved-IL-1β induced by LPS, indicating that A20 suppressed the activation of the NLRP3 inflammasome. Given that ATG5 silencing inhibited autophagy (Figure 4) and diminished the inhibitory effect of A20 on the NLRP3 inflammasome, these data demonstrate that A20’s suppression of NLRP3 inflammasome activation in BEECs is mediated through autophagy.

## 4. Discussion

During pregnancy, the vulva, vagina, cervix, and cervical mucus plug function as physical barriers to prevent pathogen invasion in cows. However, this barrier is compromised after delivery, allowing bacteria to enter the uterine cavity and colonize the endometrium, potentially causing uterine diseases [23]. LPS, a major component of Gram-negative bacterial outer membranes, triggers intense inflammatory responses upon recognition by host cell [24]. NLRP3, an intracellular pattern recognition receptor, monitors pathogen invasion and activates multiprotein inflammasome complexes, leading to Caspase-1 activation and Pro-IL-1β cleavage into its mature form. NLRP3 inflammasome hyperactivation plays crucial roles in various inflammatory diseases, including arthritis, systemic lupus erythematosus, asthma, and atherosclerosis [25,26,27]. Therefore, strict control of NLRP3 inflammasome activity is essential for maintaining homeostasis and preventing severe pathological changes.

Multiple studies have confirmed that LPS effectively activates the NLRP3 inflammasome. You et al. demonstrated that LPS induces NLRP3 inflammasome activation in human umbilical vein endothelial cells, leading to increased LDH release and elevated IL-1β expression, subsequently damaging endothelial cells [28]. Additionally, Zhao et al. verified that LPS stimulation of microglia results in decreased cell viability, increased apoptosis, and significantly elevated expression levels of NLRP3, IL-1β, and Caspase-1 [29]. Our study corroborates these findings, showing that LPS induction markedly increases cellular Caspase-1 activity and mature pro-inflammatory factor IL-1β levels. Furthermore, LDH, a characteristic marker of pyroptosis, showed significantly elevated levels in the culture medium, indicating inflammatory damage caused membrane rupture and release of cellular contents into the extracellular matrix [30,31,32]. SEM observation revealed numerous vesicular protrusions and holes in the cell membrane, further confirming LPS-induced pyroptosis in BEECs. These results support the hypothesis that LPS activates the NLRP3 inflammasome, leading to inflammatory damage in BEECs.

Research indicates that NLRP3 inflammasome activation is regulated by autophagy [33,34,35]. Autophagy activation represents a crucial innate immune response and serves as a primary defense against pathogen infection. During infection, Toll-like receptors recognize PAMPs and modulate autophagy to initiate pathogen clearance and maintain cellular homeostasis [36]. Autophagosomes capture pathogens and transport them to lysosomes for degradation, effectively eliminating pathogens while presenting pathogenic components to the immune system to promote immune responses [37,38]. Therefore, moderate enhancement of autophagy facilitates pathogen clearance efficiency. Studies have shown that ATG16L1-deficient mouse macrophages exhibit more severe increases in Caspase-1 activity and IL-1β expression when stimulated with LPS [39]. Similarly, ATG5-silenced human colonic adenoma cells show significantly elevated IL-1β production under LPS stimulation [40]. Our previous research demonstrated that LPS inhibits autophagy in BEECs, thereby increasing the expression of inflammatory mediators such as IL-6, IL-8, and TNF-α [18]. In this study, further suppression of BEECs autophagy using CQ amplified the increase in Caspase-1 activity and IL-1β expression under LPS treatment, while significantly increasing LDH release. Autophagy inhibition notably promotes NLRP3 inflammasome activation. Han et al. reported that kaempferol enhances microglial autophagy activity, promotes LC3II expression, and accelerates inflammasome component degradation, thereby inhibiting inflammasome activation [41]. After kaempferol pretreatment, NLRP3 protein levels decreased significantly, disrupting NLRP3 inflammasome assembly and reducing Caspase-1 activation and IL-1β release. NLRP3 inflammasome downregulation primarily occurs through autophagy-mediated degradation, as this phenomenon is blocked by the autophagy inhibitor 3-MA. Similarly, our research confirms that inhibiting autophagy through CQ pretreatment or ATG5 silencing significantly increases the expression of key NLRP3 inflammasome proteins. In conclusion, these results emphasize the negative regulatory role of autophagy in NLRP3 inflammasome activation, indicating that autophagy inhibition exacerbates inflammatory damage in BEECs.

A20, a deubiquitinating enzyme, functions as a self-protective molecule with anti-inflammatory properties and is closely associated with various inflammatory diseases. A20-deficient mice exhibit widespread tissue inflammation, cachexia, and even perinatal death [42,43]. Conversely, A20-overexpressing mice show reduced lupus inflammation and decreased kidney damage through inflammasome inhibition [44]. These findings confirm A20’s crucial role in preventing inflammatory responses. Our study reveals that A20 overexpression in BEECs significantly inhibits Caspase-1 activation, LDH release, and IL-1β expression. Under A20 knockdown conditions, more severe inflammatory damage occurs, demonstrating A20’s protective role against inflammatory injury in BEECs. However, the specific mechanisms by which A20 regulates inflammation in BEECs require further investigation. This study finds that A20 significantly promotes autophagy while inhibiting NLRP3 and Caspase-1 expression, suggesting regulation of inflammatory injury through the autophagy pathway. To verify whether A20 inhibits NLRP3 inflammasome-mediated inflammatory injury by promoting autophagy, we used lentiviral ATG5 shRNA to suppress A20-induced autophagy and analyzed NLRP3 inflammasome activity. Western Blot analysis shows that after ATG5 knockdown, A20’s inhibitory effects on NLRP3, Pro-Caspase-1, Cleaved-Caspase-1, Pro-IL-1β, and Cleaved-IL-1β protein expression were significantly weakened. Similarly, Tang et al. demonstrated that A20 overexpression reduces pyroptosis in human periodontal ligament cells through enhanced autophagy [45]. Zhai et al. identified that A20 directly interact with and stabilize DEPTOR via its zinc-finger domains, forming a complex that rapidly promotes autophagy upon LPS stimulation to restrain NLRP3 inflammasome activation in monocytes [46]. These findings suggest that A20 may exert anti-inflammatory effects by activating autophagy to reduce NLRP3 inflammasome activation. However, a notable limitation of the present study is that the upstream mechanism of how A20 initiates autophagy in BEECs remains unclear. It is crucial to elucidate this initial step for a complete understanding of the pathway. Therefore, future study should focus on how A20 interacts with key autophagy-initiating components to facilitate autophagosome formation.

## 5. Conclusions

**Figure 7:**
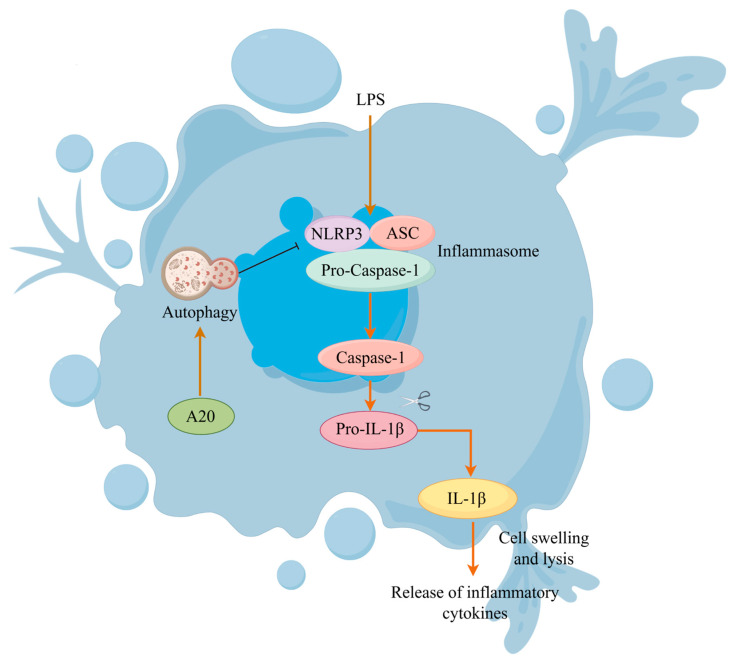
Schematic of A20 attenuating inflammatory damage in BEECs through autophagy-mediated inhibition of the NLRP3 inflammasome.

In conclusion, NLRP3 inflammasome activation plays a crucial role in LPS-induced inflammatory injury of BEECs. A20 enhances autophagy, thereby weakening LPS-induced NLRP3 inflammasome activation in BEECs and reducing inflammatory damage (Figure 7). These results suggest that A20 represents a promising therapeutic target for preventing and treating bovine endometritis, providing a novel approach for managing this condition.

## Figures and Tables

**Table 1:** Primer sequences for the target genes.

Gene	Primers (5′→3′)	Product Size (bp)	Accession Number
β-actin	F: CATCACCATCGGCAATGAGC R: AGCACCGTGTTGGCGTAGAG	156	NM_173979.3
IL-1β	F: TGATGACCCTAAACAGATGAAGAGC R: CCACGATGACCGACACCACCT	134	NM_174093.1

## Data Availability

The data presented in this study is available in the article.
